# Targeted Drug Delivery Technologies Potentiate the Overall Therapeutic Efficacy of an Indole Derivative in a Mouse Cystic Fibrosis Setting

**DOI:** 10.3390/cells10071601

**Published:** 2021-06-25

**Authors:** Matteo Puccetti, Marilena Pariano, Giorgia Renga, Ilaria Santarelli, Fiorella D’Onofrio, Marina M. Bellet, Claudia Stincardini, Andrea Bartoli, Claudio Costantini, Luigina Romani, Maurizio Ricci, Stefano Giovagnoli

**Affiliations:** 1 Department of Pharmaceutical Sciences, University of Perugia, 06123 Perugia, Italy; maurizio.ricci@unipg.it; 2 Department of Medicine and Surgery, University of Perugia, 06132 Perugia, Italy; marilena.pariano@gmail.com (M.P.); rengagiorgia@gmail.com (G.R.); ilasanta@libero.it (I.S.); donofrio.fiorella@libero.it (F.D.); marinamaria.bellet@unipg.it (M.M.B.); claudiastincardini@gmail.com (C.S.); andrea.bartoli@unipg.it (A.B.); claudio.costantini@unipg.it (C.C.); luigina.romani@unipg.it (L.R.)

**Keywords:** cystic fibrosis, inflammation, aryl hydrocarbon receptor, drug delivery, 3-IAld

## Abstract

Inflammation plays a major role in the pathophysiology of cystic fibrosis (CF), a multisystem disease. Anti-inflammatory therapies are, therefore, of interest in CF, provided that the inhibition of inflammation does not compromise the ability to fight pathogens. Here, we assess whether indole-3-aldehyde (3-IAld), a ligand of the aryl hydrocarbon receptor (AhR), may encompass such an activity. We resorted to biopharmaceutical technologies in order to deliver 3-IAld directly into the lung, via dry powder inhalation, or into the gut, via enteric microparticles, in murine models of CF infection and inflammation. We found the site-specific delivery of 3-IAld to be an efficient strategy to restore immune and microbial homeostasis in CF organs, and mitigate lung and gut inflammatory pathology in response to fungal infections, in the relative absence of local and systemic inflammatory toxicity. Thus, enhanced delivery to target organs of AhR agonists, such as 3-IAld, may pave the way for the development of safe and effective anti-inflammatory agents in CF.

## 1. Introduction

Inflammation plays a major role in the pathophysiology of cystic fibrosis (CF), a multiorgan inflammatory disease caused by mutations in the gene encoding the anion channel CF transmembrane receptor [1]. Anti-inflammatory therapies are, therefore, of interest in CF, provided that the inhibition of inflammation does not compromise the ability to fight pathogens. This demands for strategies that, mimicking endogenous homeostatic pathways, regulate inflammation, to efficiently respond to infection while preventing lung damage [2].

The aryl hydrocarbon receptor (AhR) is a ligand-dependent basic helix-loop-helix transcription factor that is highly conserved evolutionarily, and is expressed in the majority of immune cell types and human tissues [3,4]. Traditionally considered for its ability to metabolize harmful toxicants via activation of the cytochrome P450 drug-metabolizing enzymes, AhR is increasingly being recognized for its multiplicity of functions, including developmental biology and cross-talk with the microbiome for regulation of host immunity, tolerance and metabolism [3,4]. In particular, the ability of AhR to engage Th17 cells for antimicrobial activity, induce IL-22 production for epithelial cell repair and protection, and activate regulatory T cells for control of inflammation, makes the intestinal and respiratory barriers very sensitive to AhR activity and activation [5,6,7,8]. For instance, the AhR ligand ITE (2-(1H-Indol-3-ylcarbonyl)-4-thiazol carboxylic acid methyl ester), found in the mammalian lung, exhibits important immunomodulatory properties both locally and at distant sites [9]. Therefore, AhR ligands are promising compounds for pharmaceutical drug discovery, to treat inflammatory pathology at mucosal surfaces.

In addition to the prototypic AhR ligand, the toxicant 2,3,7,8-tetrachlorodibenzo-*p*-dioxin (TCDD, dioxin), pharmaceuticals, phytochemicals, such as flavonoids, and endogenous biochemicals, including indigoids, kynurenine, 6-formylindolo-[3,2-b]-carbazole and bilirubin, are among the known AhR ligands acting as selective AhR modulators with either agonist, partial agonist or antagonist activities [10,11]. More recently, a number of studies have highlighted the capacity of AhR to respond to indoles and indolyl metabolites, thus positioning AhR as a candidate indole receptor [12]. Indoles represent a wide group of gut bacteria-derived compounds produced from tryptophan, which exert significant biological effects, and may contribute to the etiology of cardiovascular, metabolic, and psychiatric diseases [13,14]. We have shown that the indole-3-carboxyaldehyde (3-IAld), abundantly produced by *Lactobacillus reuteri* in condition of trp availability [15], acts as a ligand of both murine and human AhR. Despite the uncertainty regarding the receptor activity, 3-IAld, similarly to indoles, moonlights as a metabolite and signaling molecule, and is increasingly being associated with the regulation of wide-ranging physiological processes [16]. However, the majority of the research in the field is limited to experimental studies, likely accounted for by the context-and ligand-dependent activity of AhR [5,17]. Therefore, the activation of AhR holds great therapeutic potential, but research is needed to convey this potential towards a reproducible and optimal efficacy/safety profile, and the application of molecular pharmaceutics might represent a turning point in this direction. Indeed, the possibility of resorting to suitable biopharmaceutical formulations to enable site-specific drug delivery is urgently needed to improve therapeutic efficacy, decrease unwanted toxicities and prevent off-target effects. In this regard, the complex symptomatology of CF would greatly benefit from a targeted drug delivery that, by reverting the chronic inflammatory state and protecting from recurrent infections, restores organ physiology without causing alterations at other sites.

In the present study, we have resorted to spray-drying techniques to produce inhalable dry powders for lung delivery and enteric microparticles for intestine release, to assess the efficacy of 3-IAld in a murine model of CF infection and inflammation in the lung and gut. We found that site-specific delivery of 3-IAld is an efficient strategy to mitigate lung and gut inflammatory pathology, by restoring immune and microbial homeostasis in CF organs. This study paves the way for the development of safe and effective AhR agonists as anti-inflammatory agents in CF via improved delivery to target organs.

## 2. Materials and Methods

### 2.1. Mice and Treatments

Murine experiments were performed according to the Italian Approved Animal Welfare Authorizations 360/2015-PR and 396/2018 PR, lasting for five years (2015–2020) and two years (2018–2020), respectively, and Legislative Decree 26/2014 regarding the animal license, obtained by the Italian Ministry of Health. Four- to six-week-old C57BL/6 mice were purchased from Charles River (Calco, Italy). Mouse were housed in a controlled environment at the Animal Facility of Perugia University and provided with standard rodent chow and water. CF mice homozygous for the F508del-CFTR (referred to as *Cftr ^F508del/F508del^* mice), which had been backcrossed for 12 generations to the C57BL/6 strain, or in the FVB/129 outbred background (*Cftr ^tm1EUR^, F508del*), were obtained from Bob Scholte, Erasmus Medical Center Rotterdam, the Netherlands [18]. For *Aspergillus* infection, mice were anesthetized in a plastic cage by inhalation of 3% isoflurane (Forane, Abbott) in oxygen before intranasal instillation of 2 × 10^7^ *Aspergillus fumigatus* (Af293) resting conidia per 20 μL of saline. The 3-IAld was delivered intranasally (20 μL), by liquid aerosol, and orally (3-IAld-MP in the Eudragit formulation, [19]) at a dose of 18 mg/kg, or by pulmonary insufflation (3-IAld-PI), at a dose of 4.5 mg/kg using a dry powder insufflator model DP-4M (Penn-Century Inc., Wyndmoor, PA, USA). Mice were treated with 3-IAld 3 times either the week before (prophylactic treatment) or the week after (therapeutic treatment) the infection, except for the pulmonary insufflation where it was administered once 3 days before or after the infection. For *Candida albicans* infection, 1 × 10^8^ *C. albicans* SC5314 cells were given intragastrically in 200 μL saline using an 18G 4-cm-long plastic catheter. Mice received 18 mg/kg 3-IAld-MP orally 3 times the week before the infection. Mice were sacrificed 7 days after the infection. Lungs of mice were lavaged three times with 0.5 mL of PBS via the tracheal tube, and bronchoalveolar lavage fluid (BAL) was stored at −80 °C until use. Fungal growth was expressed as colony-forming units (Log_10_ CFU) per organ, obtained by serially diluting homogenates on Sabouraud agar plates incubated at 37 °C for 24 h. Untreated mice received the vehicle alone, empty Eudragit or mannitol for the oral or pulmonary insufflation, respectively. Polymorphonuclear cells (PMN) recruitment in the bronchoalveolar lavage fluids (BAL) was assessed on cytospin slides, and inflammatory cytokine gene expression and production was assessed by RT-PCR and ELISA, respectively. For histology, tissues were removed and fixed in 10% phosphate-buffered formalin (Bio Optica) and stained with periodic acid–Schiff (PAS); for immunofluorescence, paraffin-embedded sections were rehydrated and, after antigen retrieval in citrate buffer (10 mM, pH 6), fixed in 2% formaldehyde for 40 min at room temperature and permeabilized in a blocking buffer containing 5% FBS, 3% BSA, and 0.5% Triton X-100 in PBS. The slides were then incubated at 4 °C with primary antibodies anti–Ki67 (Abcam, ab15580, 5 µg/mL) and anti-ZO-1 (ThermoFisher, 61-7300, 5 µg/mL). After extensive washing with PBS, the slides were then incubated at room temperature for 60 min with secondary antibodies, anti-rabbit DyLight550 (BETHYL) and anti-rabbit Alexa Fluor 488 (Molecular Probe). Nuclei were counterstained with DAPI. Images were acquired using a microscope BX51 and analySIS image processing software (Olympus).

### 2.2. Inhalable 3-IAld Dry Powder Preparation

Spray-dried formulation of 3-IAld, containing mannitol as excipient at 2:1 *w*/*w* ratio, was prepared using a mini spray-dryer model B-290 (Büchi, Switzerland). Solution of 3-IAld:mannitol was prepared in water/ethanol (50:50 *v*/*v*). Spray-drying was performed, in co-current mode, by a spray-dryer equipped with a 2-fluid nozzle having a 0.7 mm nozzle tip and a 1.5 mm diameter nozzle cap. The operating spray drying parameters were as follows: inlet temperature 75 °C, air flow rate 301 L/h, 2.4 mL/min feed rate and aspirator rate of 20 m^3^/h. Briefly, 3-IAld was dispersed in the ethanol solvent and allowed to solubilize under magnetic stirring before adding into mannitol water solution. The obtained dried powders were recovered by using a high-performance cyclone (Büchi, Switzerland). 

### 2.3. Enteric Formulation Preparation

The enteric MPs were prepared using Eudragit^®^ L100-to-S100 (Rohm Pharma GmbH, Darmstadt, Germany) ratio of 1:2 with the addition EC (30% *w*/*w*, ETHOCEL std. 7, Dow Chemical Company, Milan, Italy) as described [19]. The 3-IAld (Sigma-Aldrich, Milan, Italy) and the polymers were dissolved in ethanol at a feedstock concentration of 3% *w*/*v* and spray-dried, at the inlet temperature of 75 °C, using a mini spray-dryer model B-290 (Büchi, Italy) in co-current mode, equipped with a two-fluid nozzle having a 0.7 mm nozzle tip and a 1.5 mm diameter nozzle cap. Aspirator capacity was maintained at 20 m^3^/h, air flow rate was 301 L/h, and feed rate 2.4 mL/min. The obtained dried MP were recovered by using a high-performance cyclone (Büchi, Milan, Italy).

### 2.4. Ex Vivo Organ Cultures

We cultured ex-vivo organs from untreated mice in 2 mL RPMI-1640 medium containing 10% heat-inactivated FBS (Gibco, Invitrogen, Milan, Italy), 100 U mL^–1^ penicillin, 100 g mL^–1^ streptomycin and 10 mM HEPES (Gibco, Invitrogen) with 3-IAld (Sigma-Aldrich) at different concentrations for 24 h before cytokine determination by ELISA in the culture supernatants (without dilution) and by RT-PCR in recovered cells.

### 2.5. RNA Extraction and Quantitative RT-PCR (qRT-PCR)

Total RNA was extracted from lungs by the TRIzol method (Invitrogen, Milan, Italy) according to the manufacturer’s protocol. The PrimeScript RT reagent kit with gDNA Eraser (Takara, Japan) was used for reverse transcription according to the manufacturer’s protocol. A real-time PCR amplification (PCR using CFX96 Touch™ Real-Time PCR Detection System) was performed with iTaq™ Universal SYBR^®^ Green Supermix (Bio-Rad, Milan, Italy) under the following conditions: 45 cycles of 95 °C for 1 min, appropriate annealing temperature for 1 min, and 72 °C for 30 s. All reactions were repeated at least three times independently to ensure the reproducibility of the results. Primers used are reported in Supplementary Table S1.

### 2.6. ELISA Assay

Cytokine content was determined by enzyme-linked immunosorbent assays on tissue homogenates. Briefly, ELISA plates were coated with 50 μL of anti-cytokine capture antibody at 4 °C overnight. Plates were then washed 3× with 0.05% PBS-Tween (PBST) and coated for 1 h with the 150 μL 1% BSA/PBS blocking buffer. Samples or standards were added in duplicates (50 μL per well) and incubated for 2 h at room temperature. Wells were washed 3× with PBST and incubated with 50 μL of anti-cytokine detection antibody at 4 °C overnight. Wells were then washed 3× with PBST and incubated with 50 μL of avidin-HRP at room temperature for 30 min. Thereafter, wells were washed × 5 with PBST and incubated with 50 μL per well of a substrate. The reaction was stopped after 15 min with 1 M H_2_SO_4_ and absorbance was measured using a TECAN microplate fluorescence reader (Infinite M200) at 405 nm and 570 nm.

### 2.7. Bacterial DNA Extraction and Quantitative PCR for Microbiota Analysis

Bacterial DNA from feces of mice was extracted using a QIAamp DNA stool mini kit (Qiagen). Bacteria species-specific PCR was carried out as described [20] with primers targeted on the 16S rRNA using CFX96 Touch™ Real-Time PCR Detection System and iTaq™ Universal SYBR^®^ Green Supermix (Bio-Rad, Milan, Italy). The amplification program was 45 cycles of 95 °C for 1 min, appropriate annealing temperature for 1 min, and 72 °C for 30 s. Bacterial abundances were expressed as relative 16S rRNA gene levels. Primers used are reported in Supplementary Table S1.

### 2.8. Statistical Analysis

GraphPad Prism software 6.01 (GraphPad, San Diego, CA, USA) was used for the analysis. Data are expressed as mean ± SD. Statistical significance was calculated by one-way ANOVA (Tukey’s or Bonferroni’s post hoc test) for multiple comparisons and by a two-tailed Student’s *t*-test for single comparison. We considered all *p*-values < 0.05 significant. The in vivo groups consisted of 4–6 mice/group. Data represent pooled results or are representative of 3 experiments.

## 3. Results

### 3.1. 3-IAld Inhalable Powder Protects Cftr ^F508del/F508del^ from Lung Pathology

**Figure 1:**
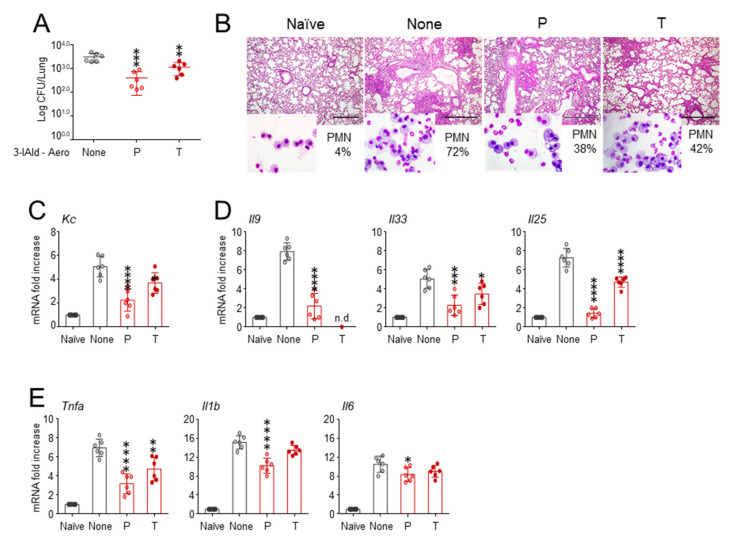
The 3-IAld administered by aerosolization prevents lung inflammatory pathology in CF. *Cftr ^F508del/F508del^* mice were infected with live *A. fumigatus* conidia and treated with 18 mg/kg of 3-IAld three times the week before (prophylactic, P) or after (therapeutic, T) the infection. (**A**) Fungal growth (Log_10_ CFU in the lung); (**B**) PAS staining of the lung and % neutrophil recruitment in the bronchoalveolar lavage (insets); expression of neutrophil-recruiting chemokine (**C**) and cytokines (**D**,**E**) genes by RT-PCR in the lung. Assays were done 7 days after the infection. Photographs were taken using a high-resolution Olympus DP71 microscope using a 10× objective. Scale bar 400 μm. Values represent the mean ± SD of six mice per group or are representative of three experiments. Naïve, uninfected mice. None, infected and untreated mice. *, *p* < 0.05; **, *p* < 0.01; ***, *p* < 0.001; ****, *p* < 0.0001, one-way ANOVA—Bonferroni’s, P or T vs. None.

In a set of preliminary experiments, in which 3-IAld, at different doses, was administered intranasally in C57BL/6 mice infected with *Aspergillus* conidia, we found that the dose of 18 mg/kg (0.36 mg/mouse) significantly reduced fungal growth, and the effect was not increased at the higher doses (Supplementary Figure S1), which is in agreement with previous findings [21]. Therefore, to evaluate whether the therapeutic activity of 3-IAld would extend to similarly infected *Cftr ^F508del/F508del^* mice, the dose corresponding to 18 mg/kg was administered by aerosolization of a 3-IAld physiologic solution. The mice were monitored for local fungal growth, lung histopathology, inflammatory cell recruitment in the BAL, and cytokine/chemokine gene expression. We found that 3-IAld aerosolized either before (P) or after (T) fungal challenge exerted significant control on the infection and the ensuing inflammatory response. Indeed, the treatment reduced the fungal growth (Figure 1A) and inflammatory pathology (Figure 1B) in the lung, reduced the neutrophils recruitment in the BAL (insets of Figure 1B) and the expression of neutrophil-recruiting chemokine (Figure 1C). The treatment also reduced the expression of cytokines known to be involved in epithelial dysfunction and mucosal immune dysregulation, such as *Il9*, *Il25* and *Il33* (Figure 1D) [22]. The prototypal inflammatory cytokine expression, such as *Tnfa*, *Il1b* and *Il6*, were also significantly reduced (Figure 1E).

**Figure 2:**
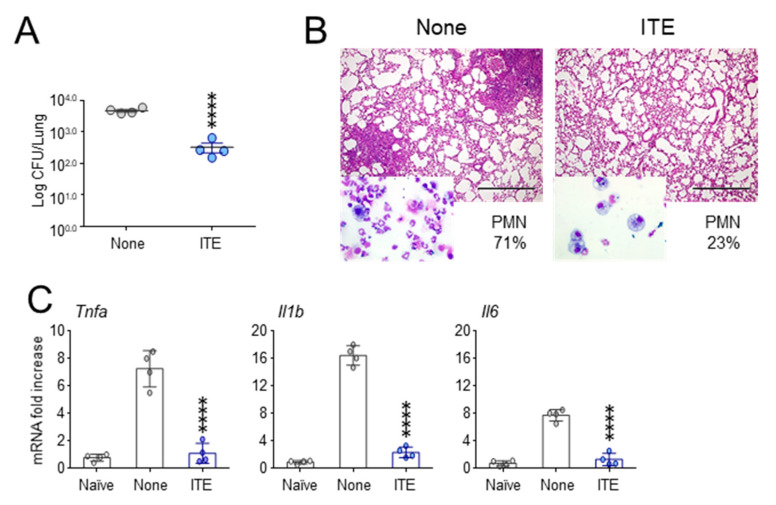
The AhR ligand ITE prevents lung inflammatory pathology in CF. *Cftr ^F508del/F508del^* mice were infected with live *A. fumigatus* conidia and treated with 10 mg/kg of ITE intraperitoneally three times the week before the infection. (**A**) Fungal growth (Log_10_ CFU in the lung); (**B**) PAS staining of the lung and polymorphonuclear cells (PMN) recruitment (%) in the bronchoalveolar lavage (insets); (**C**) expression of inflammatory cytokine genes in the lung by RT-PCR. Assays were done 7 days after the infection. Photographs were taken using a high-resolution Olympus DP71 microscope using a 10× objective. Scale bar 400 μm. Values represent the mean ± SD of four mice per group or are representative of three experiments. Naïve, uninfected mice. None, infected mice. **** *p* < 0.0001, Student *t*-test and one-way ANOVA—Bonferroni’s, ITE-treated vs. None.

Because in the same experiment, treatment with the AhR ligand ITE more effectively controlled the fungal growth (Figure 2A), lung inflammation and neutrophils recruitment in the BAL (Figure 2B and insets), and, importantly, significantly decreased the expression of inflammatory *Tnfa*, *Il1b* and *Il6* cytokines (Figure 2C), this prompted us to assess whether a more consistent dose delivery would improve the efficacy of 3-IAld.

**Figure 3:**
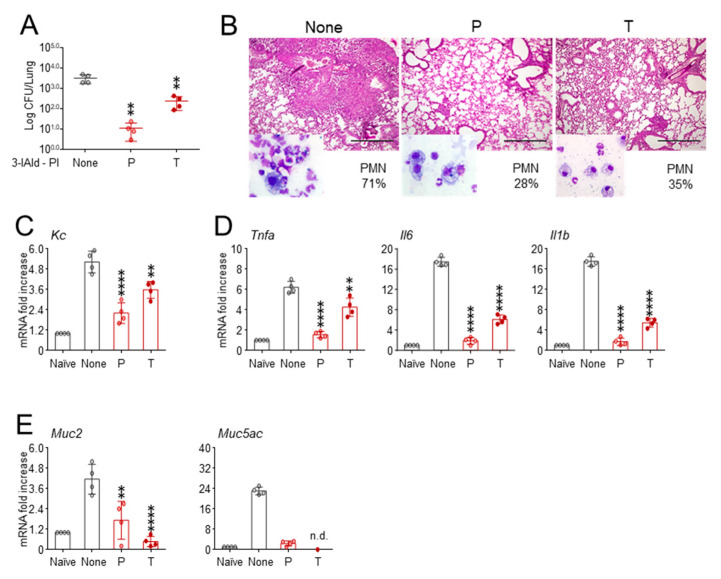
Pulmonary insufflation of 3-IAld prevents lung inflammatory pathology in CF. *Cftr ^F508del/F508del^* mice were infected with live *A. fumigatus* conidia and treated with 4.5 mg/kg of 3-IAld directly delivered into the lung by pulmonary insufflation (PI), either 3 days before (P, prophylaxis) or after the infection (T, therapy). (**A**) Fungal growth (Log_10_ CFU in the lung); (**B**) PAS staining of the lung and neutrophil recruitment (%) in the bronchoalveolar lavage (insets); gene expression of PMN-recruiting chemokine (**C**), pro-inflammatory cytokine (**D**) and *Muc2* and *Muc5ac* (**E**) by RT-PCR. Assays were done 7 days after the infection. Photographs were taken using a high-resolution Olympus DP71 microscope using a 10× objective. Scale bar 400 μm. Values represent the mean ± SD of four mice per group or are representative of three experiments. Naïve, uninfected mice; None, infected mice. ** *p* < 0.01; **** *p* <0.0001, one-way ANOVA—Bonferroni’s, P or T vs. None. n.d, not done.

To this purpose, we developed an inhalable dry powder of 3-IAld that could potentially allow deposition in the lower respiratory tract [23]. To avoid distress, due to the insufflation of an excessive mass of powder, and to ensure dose reproducibility, we delivered the 3-IAld inhalable powder (3-IAld-IP) at 4.5 mg/kg, one-fourth of the oral dose, by insufflation to infected *Cftr ^F508del/F508del^* mice, once, either before or after the infection. The results show that prophylactic, more than therapeutic, administration of 3-IAld-IP greatly inhibited local fungal growth (Figure 3A), ameliorated peribronchial inflammatory pathology (Figure 3B), reduced neutrophils in the BAL (insets of Figure 3B), and neutrophil-recruiting chemokine expression (Figure 3C). Concomitantly, the level of expression of pro-inflammatory cytokine genes was significantly reduced (Figure 3D). Of interest, 3-IAld-IP also reduced the expression of *Muc2* and *Muc5ac* genes (Figure 3E), both of which are known to reflect goblet cells hyperplasia in the CF airways [24]. No signs of tissue toxicity nor activation of AhR target genes were observed in the distant organs (Supplementary Figure S2).

These results suggest that the targeted delivery of 3-IAld could be of therapeutic value in preventing lung infection and inflammation in CF, at a reduced dose and without systemic activity.

### 3.2. Enteric Formulated 3-IAld Protects Cftr ^F508del/F508del^ from Gut Pathology

**Figure 4:**
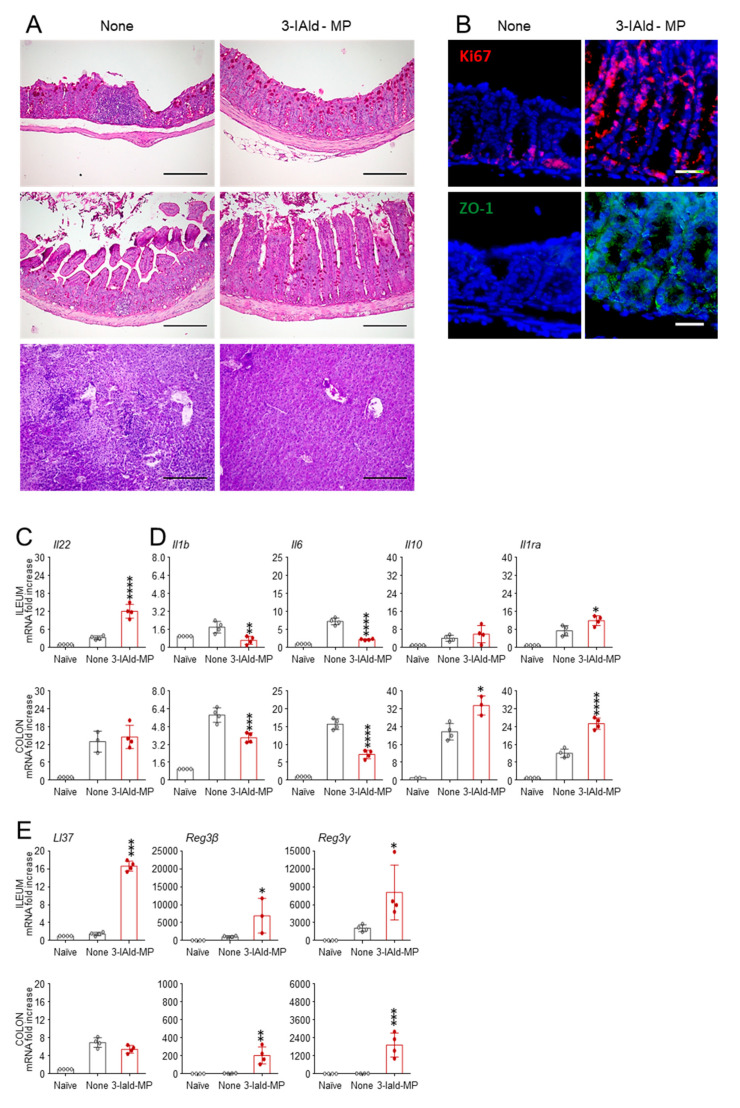
Enteric formulated 3-IAld protects from gut pathology in CF. *Cftr ^F508del/F508del^* mice were infected with *C. albicans* and treated with 18 mg/kg of 3-IAld-MP given orally three times the week before the infection. (**A**) PAS staining of the colon, ileum and liver; (**B**) expression of Ki67 and ZO-1 in the colon; expression of (**C**) *Il22*; (**D**) inflammatory and anti-inflammatory cytokines; (**E**) antimicrobial peptide *LL37* and defensins by RT-PCR in the ileum and colon. Assays were done 7 days after the infection. For immunofluorescence, nuclei were counterstained with DAPI. Photographs were taken using a high-resolution Olympus DP71 microscope using an 20× objective. Scale bar 200 μm. Values represent the mean ± SD of four mice per group or are representative of three experiments. Naïve, uninfected mice; None, infected mice. * *p* < 0.05; ** *p* < 0.01; *** *p* < 0.001; **** *p* < 0.0001, one-way ANOVA—Bonferroni’s, 3-IAld-MP vs. None.

Consistent with the gut abnormalities observed in CF [25], *Cftr ^F508del/F508del^* mice are highly susceptible to gastrointestinal infection with the fungus *C. albicans* [20]. We resorted to CF mice with gastrointestinal *C. albicans* infection as a reference model, to assess whether, similar to what was observed in the lung, 3-IAld exert a beneficial effect in gut inflammatory pathology. To this purpose, we administered infected mice with 3-IAld encapsulated in enteric microparticles (3-IAld-MP), a formulation that we have recently shown to prevent the metabolic and immune complications associated with impaired intestinal epithelial barrier function in the relative absence of local and distal immunotoxicity [21]. Consistent with the results obtained in wild-type mice [15], the treatment with 3-IAld-MP, while it did not significantly affect the local fungal growth (data not shown), it greatly improved the local barrier function and tissue inflammation. The cross-sections examined for general tissue morphology (PAS), epithelial proliferation and renewal (Ki67), and epithelial tight junction expression (ZO-1) revealed that 3-IAld-MP restored epithelial architecture in the ileum and colon (Figure 4A), promoted proliferation and restored tight junction structures (Figure 4B), likely by increasing the local production of IL-22, which is a cytokine involved in the restoration of barrier function at mucosal surfaces [26] (Figure 4C). On histological evaluation, 3-IAld-MP ameliorated the signs of tissue pathology in these organs (Figure 4A), and, of interest, also in the liver, (Figure 4A) a finding indicating that *Candida* dissemination to visceral organs was prevented by 3-IAld-MP. Concomitantly, the expression of the inflammatory cytokines *Il1b* and *Il6*, and of the anti-inflammatory *Il10* and *Il1ra*, was decreased or increased, respectively, upon treatment (Figure 4D). Consistent with the restoration of the epithelial function, 3-IAld-MP induced the expression of antimicrobial peptides, which are known to play an important role in determining the outcome of the host–pathogen interaction at the mucosal confrontational sites [27], such as the cathelicidin-class antimicrobial peptide LL-37 and the Reg3 lectins (Figure 4E).

Altogether, these data indicate that 3-IAld-MP can mitigate signs of intestinal and hepatic inflammatory disease in *Cftr ^F508del/F508del^* mice.

### 3.3. Enteric Formulated 3-IAld Protects Cftr ^F508del/F508del^ Mice from Lung Pathology

**Figure 5:**
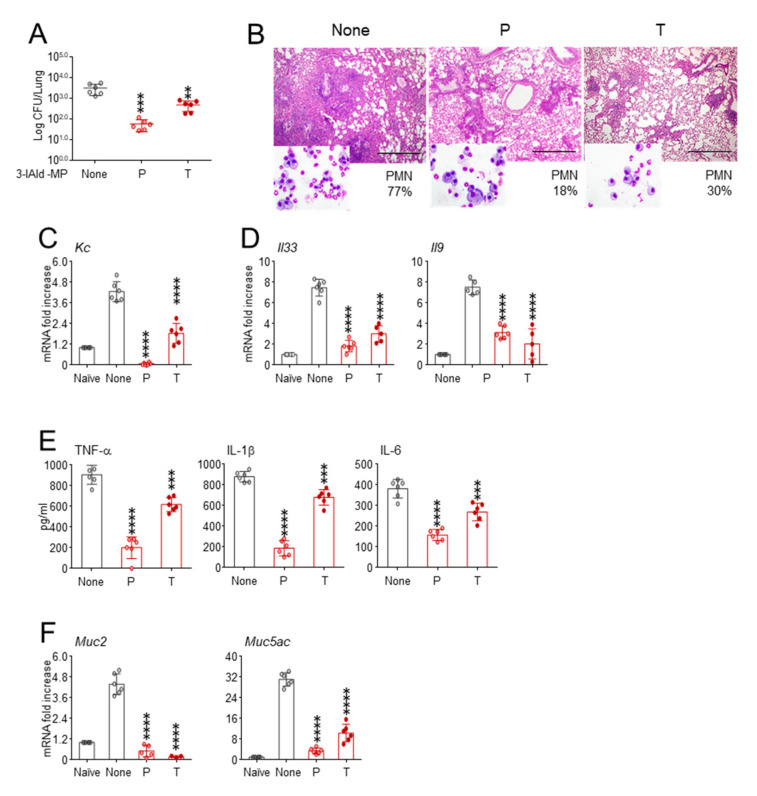
Enteric formulated 3-IAld ameliorates lung pathology in CF. *Cftr ^F508del/F508del^* mice were infected with live *A. fumigatus* conidia and treated with an oral formulation of 3-IAld (3-IAld-MP) three times, either the week before (P, prophylaxis) or after the infection (T, therapy). (**A**) Fungal growth (Log_10_ CFU in the lung); (**B**) PAS staining of the lung and PMN recruitment in the bronchoalveolar lavage (insets); gene expression of (**C**) neutrophil-recruiting chemokine and (**D**) *Il33* and *Il9* cytokines by RT-PCR; (**E**) inflammatory cytokine levels by ELISA and (**F**) expression of *Muc2* and *Muc5ac* gene by RT-PCR. Assays were done at the end of treatment. Photographs were taken using a high-resolution Olympus DP71 microscope using an 10× objective. Scale bar 400 μm. Values represent the mean ± SD of six mice per group or are representative of three experiments. None, infected mice. ** *p* < 0.01; *** *p* < 0.001; **** *p* <0.0001, one-way ANOVA—Bonferroni’s, P or T vs. None.

The above results indicate that 3-IAld exert multiple and important therapeutic effects in CF upon appropriate delivery in the inflamed organs. However, to gain further preclinical insights that will be relevant for future clinical developments, we have also assessed whether the activity of orally delivered 3-IAld would extend to the lung. To this purpose, *Cftr ^F508del/F508del^* mice were infected with *Aspergillus* conidia, treated with 3-IAld–MP three times a week for a week, either before or after the infection, and assessed for parameters of infection and inflammation. The results show that prophylactic, more than therapeutic, administration of 3-IAld-MP inhibited local fungal growth (Figure 5A), greatly ameliorated peribronchial inflammatory cell recruitment in the lung (Figure 5B), reduced neutrophils in the BAL (insets of Figure 5B) and neutrophil-recruiting chemokine expression (Figure 5C). Concomitantly, the level of expression of *Il33* and *Il9* (Figure 5D), *Tnfa*, *Il1b*, and *Il6* (Figure 5E), and of *Muc2* and *Muc5ac* genes (Figure 5F), were all significantly reduced.

These results point to the therapeutic utility of 3-IAld in preventing lung infection and inflammation via the intestinal mucosa.

### 3.4. 3-IAld Activates AhR in Ex-Vivo Organ Cultures

**Figure 6:**
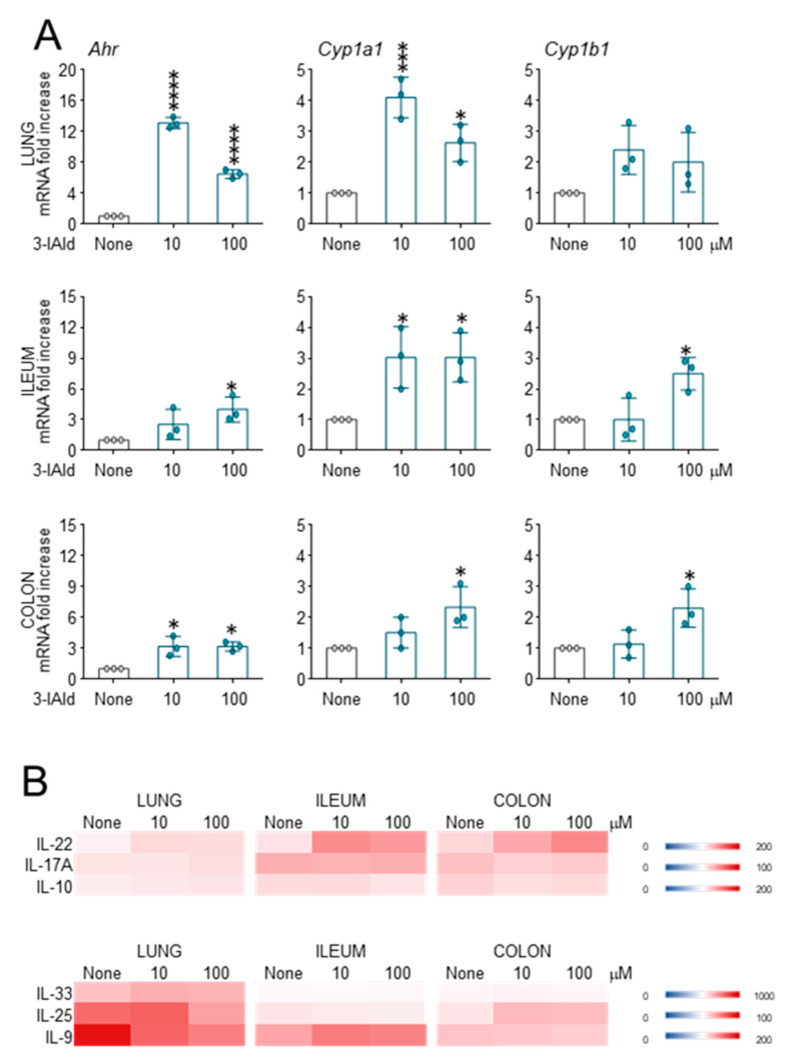
The 3-IAld activates AhR in ex vivo organ cultures. Assessment of gene expression (**A**) and cytokine determination (**B**) in lung, ileum and colon from naïve mice exposed to 3-IAld for 24 h in vitro. Cytokines were quantified by ELISA in the culture supernatants (pg/mL) and gene expression by RT-PCR in recovered cells. None, untreated organs. Data represent pooled results (n = 3, mean ± SD). * *p* < 0.05; *** *p* < 0.001; **** *p* < 0.0001, one-way ANOVA—Bonferroni’s, treated vs. None.

The 3-IAld is known to act as an AhR agonist, both in vitro and in vivo in the gut and lung of immunocompetent mice. To corroborate these findings, we resorted here to an organ culture system in vitro, to assess the dose-dependent activity of 3-IAld in an unbiased environment, mimicking the entire organ in vivo. To this purpose, lung, ileum and colon from naïve mice were exposed to 3-IAld for 24 h before gene expression assessment and cytokine determination in the supernatants. We evaluated the influence of 3-IAld on the expression and activity of AhR and AhR-dependent genes, as well as on the production of IL-9, IL-25 and IL-33. We found that 3-IAld, at doses ranging from 10 to 100 µM, activate the expression of AhR and the downstream genes *Cyp1a1* and *Cyp1b1*, both in the lung and gut (ileum and colon) (Figure 6A), and increased the levels of IL-22 being actually produced at these sites (Figure 6B). Despite the fact that some AhR ligands could also stimulate the production of cytokines, such as IL-17A and IL-10 [5,28], we did not observe the production of these cytokines in the lung or gut cultures (Figure 6B) and we did not observe the production of IL-33, IL-25 and IL-9 either (Figure 6B), which is a finding confirming the ability of 3-IAld to selectively activate AhR for epithelial barrier function [16].

### 3.5. 3-IAld Affects the Microbial Composition in the Gut and Lung

**Figure 7:**
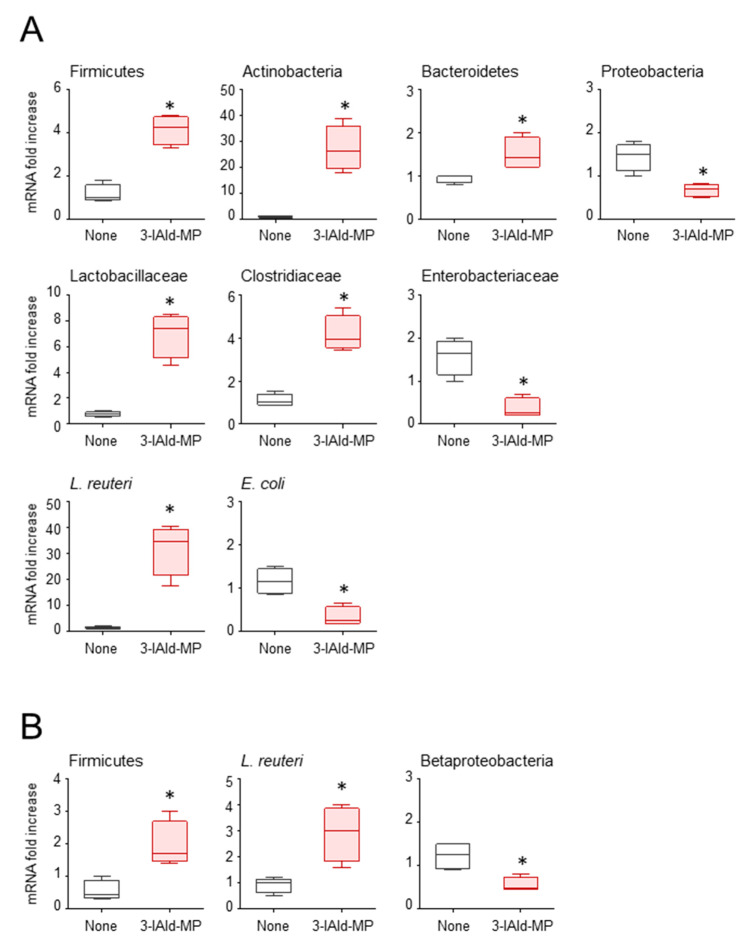
3-IAld affects microbial composition in the gut and lung. Relative abundance (by RT-PCR) of bacteria at the phylum, family and species level in the feces (**A**) and lung (**B**) of *Cftr ^F508del/F508del^* mice infected with *C. albicans* and treated with 3-IAld-MP as in legend to Figure 4. Data represent pooled results (n = 3, mean ± SD). * *p* < 0.05, Student’s *t*-test, 3-IAld-MP vs. None.

Evidence indicates that indoles produced by the gut microbiota act as a trans-kingdom signaling molecules that are capable of affecting bacterial virulence and pathogenesis [29]. We have analyzed the microbial composition in the gut and airways of *Cftr ^F508del/F508del^* mice, upon treatment with 3-IAld-MP. The results obtained by RT-PCR of feces and lung tissues showed that 3-IAld-MP significantly modified the fecal bacterial composition at the phylum level, by promoting the expansion of Firmicutes, Actinobacteria and Bacteroidetes, and significantly reducing Proteobacteria; at the family and species levels, 3-IAld-MP promoted the expansion of Lactobacillaceae (*Lactobacillus reuteri* species) and prevented the expansion of Enterobacteriaceae (*Escherichia coli* species) (Figure 7A). Of interest, Firmicutes and *L. reuteri* also expanded in the lung where beta-proteobacteria were instead restrained (Figure 7B).

As the dysbiotic expansion of Enterobacteriacae is associated with a worse outcome in children with CF [30,31], and contributes to infectious pathology in the lung [32], preliminary as they are, these data indicate a specific 3-IAld-dependent regulation of local microbial composition that could be of benefit in CF. Moreover, the expansion of *L. reuteri*, known to provide epithelia barrier function at mucosal surfaces [33], points to the unique ability of 3-IAld to dually act at the microbe/host interface.

## 4. Discussion

Despite clear improvements in CFTR function and clinical endpoints, the current evidence suggests that CFTR modulators may not prevent a continued decline in lung function, halt disease progression, or ameliorate pathogenic inflammation and its long-term consequences [34,35]. This emphasizes the need to prioritize complementary anti-inflammatory treatments in the CFTR modulators era. However, the long-term use of the anti-inflammatory drugs currently approved in CF, such as corticosteroids and nonsteroidal anti-inflammatory drugs, is often accompanied by off-target and dose-dependent effects [2]. There is a significant clinical unmet need to define new classes of safe and effective anti-inflammatory therapies via enhanced delivery to the target organ. This study provides a proof-of-concept demonstration that harnessing endogenous anti-inflammatory pathways, via enhanced targeted delivery of 3-IAld, could be a safe and effective therapeutic strategy to curb inflammation and its consequence in CF. Although the current body of literature regarding the AhR demonstrates the complex, and often contradictory, nature of this signaling pathway, much evidence supports that the AhR is necessary for the maintenance of lung and gut health [6,7,8,36]. Accordingly, this study shows that targeting AhR with a selective ligand modulator improves mucosal inflammation, strengthens epithelial barrier function, and provides antimicrobial resistance in CF mice. Similar to what has been observed upon the delivery of enteric formulated 3-IAld [21], the delivery of a 3-IAld inhalable powder was not associated with signs of toxicity to other organs, which is a finding confirming the efficacy and safety of AhR druggability in target organs via the appropriate ligands. One limitation of our study is the less severe airway phenotype of the murine models compared to the CF patients. Further studies will be required to determine the optimal protocol for the delivery of 3-IAld inhalable powder in the presence of the mucus obstruction characteristic of the disease. Notwithstanding this limitation, the results presented in this study clearly demonstrate that it is possible to resort to molecular pharmaceutics for the local delivery of an AhR agonist, to improve the efficacy/safety profile in the complex and multi-organ disease CF, thus counteracting infections and ameliorating the chronic inflammatory environment without causing deleterious side effects. With regards to the safety, 3-IAld is reminiscent of the selective AhR modulators and rapidly metabolized AhR ligands, which are emerging receptor agonists that bypass AhR-related toxicity in favor of therapeutic effects [37]. We have evidence that 3-IAld, similar to ITE, is rapidly metabolized and likely non-toxic (data not shown), as opposed to the persistent and highly toxic TCDD [38]. This likely results from the ability of 3-IAld to activate AhR-dependent detoxifying CYP-P450 enzymes in vivo [21,23] and in vitro (this study), which, by promoting the cytosolic degradation of AhR agonists, allows for a fine regulation of AhR transcriptional activity [39,40].

Although AhR may affect immunity and inflammation by acting through the different branches of the innate and adaptive immune system [3], an interesting observation of the present study points to AhR agonists as capable of affecting inflammation indirectly, through an action on the local microbiota composition. Consistent with the ability of AhR to mediate the host–microbiota interplay [41], AhR-deficient mice have indeed an increased abundance of Verrucomicrobia and segmented filamentous bacteria in the gut, which is directly linked to elevated intestinal inflammation and promotion of Th17 differentiation [42]. We found that 3-IAld affected the airway and gut microbial composition in CF mice by decreasing the expansion of Proteobacteria, which are known to contribute to inflammatory pathology in CF [43], while increasing the expansion of Firmicutes and Bacteroidetes. This finding suggests that the control of the microbial composition and fitness could be an additional mechanism through which 3-IAld may exert its therapeutic activity at the host/microbe interface. In an in vitro study, we have shown that 3-IAld exhibited potent antimicrobial activity against the relevant respiratory pathogens in CF, such as *Staphylococcus aureus* and *Pseudomonas aeruginosa* [44]. These observations indicate that 3-IAld influences the composition of airways and gut microbiota, and may exert some degree of antimicrobial activity against CF pathogens. The finding that indole and indolyl compounds function as intra- and inter-species signaling molecules across bacterial populations, affecting virulence and antibiotic resistance, has already been reported [45]. Should these observations be confirmed in future human studies, 3-IAld, similarly to CFTR modulators [46], may be endowed with the ability to modify the mucosal milieu to favor a healthier microbiota, which could lead to improvements in pulmonary and extra-pulmonary CF morbidity. Given the existence of the gut–lung axis, involving host–microbe as well as microbe–microbe interactions [47], this may account for the ability of 3-IAld to exert localized, but also long-reaching, effects upon oral administration, and perhaps offers a plausible explanation of the superior activity of 3-IAld given prophylactically, rather than therapeutically. This explanation is consistent with the notion that the interactions between the host immune system and intestinal commensal bacteria shape immune system development beyond the local environment, to include extra-intestinal sites such as the lungs. It has indeed been shown that group three innate lymphoid cells, which are targeted by 3-IAld for mucosal homeostasis [15], promote antimicrobial resistance in the lung after migration from the intestinal site [48]. We are currently working on elucidating molecular pathways by which the anti-inflammatory and antimicrobial activities of 3-IAld are at the maximum upon local administration, and how to increase the efficacy upon administration in overt disease.

The off-target effects of systemically administered drugs have been a major hurdle in designing therapies with desired efficacy and acceptable toxicity. Smart drug carrier vehicles, able to deliver medications in a manner that increases the concentration of therapeutic agents in a specified target site, potentially surpass this limitation. The potential to tackle and overcome historical challenges associated with the more classical therapies and modes of administration has opened new therapeutic avenues, mostly exploited in cancer management [49]. This study shows the feasibility of local therapies through enhanced drug delivery in chronic inflammatory diseases, such as CF, and points to the ligand-selective modulation of AhR as an attractive therapeutic approach.

## Supplementary Materials

The following are available online at https://www.mdpi.com/article/10.3390/cells10071601/s1, Table S1: list of primers; Figure S1: dose-dependent activity of 3-IAld in vivo; Figure S2: lack of systemic toxicity and AhR activation in *Cftr ^F508del/F508del^* mice administered with 3-IAld-PI.
