# Supplementary material for: Targeted Drug Delivery Technologies Potentiate the Overall Therapeutic Efficacy of an Indole Derivative in a Mouse Cystic Fibrosis Setting
Source: Cells. 2021 Jun 25;10(7):1601. doi: 10.3390/cells10071601 (PMC8305708; doi:10.3390/cells10071601)
Supplement: Supplementary file 1 [file cells-10-01601-s001.zip › cells-1219116-supplementary.pdf]

Table S1. List of primers.

**Murine**	
β-actin	forward AGCCATGTACGTAGCCATCC, reverse CTCTCAGCTGTGGTGGTGAA
Cyp1a1	forward ACAGTGATTGGCAGAGATCG, reverse GAAGGGGAC-GAAGGATGAAT
Cyp1b1	forward TTCTCCAGCTTTTTGCCTGT, reverse TAATGAAGCCGTCCTTGTCC
Il10	forward GAGAAGCATGGCCCAGAAATCAAG, reverse ATCACTCTTCAC-CTGCTCCACTGC
Il1b	forward TGACGGACCCCAAAAGATGAAGG, reverse CCACGGGAAAGA-CACAGGTAGC
Il1ra	forward TTGTGCCAAGTCTGGAGATG, reverse CAGCTGACTCAAA-GCTGGTG
Il22	forward CTGCCTGCTTCTCATTGCCCTGTG, reverse GATGTACGGCTGCTG-GAAGTTGG
Il25	forward ACCACAACCAGACGGTCTTC, reverse ACACACACACAA-GCCAAGGA
Il33	forward TCCTGCCTCCCTGAGTACAT, reverse CACCTGGTCTTGCTCTTGGT
Il6	forward CCGGAGAGGAGACTTCACAG, reverse TCCACGATTTCCCAGA-GAAC
Il9	forward TGACCAGCTGCTTGTGTCTC, reverse GTGGCATTGGTCAGCTG-TAA
Ll37	forward GGCGGTCACTATCACTGCTGCTG, reverse TCACTCGGAAC-CTCACAGACTTGG
Muc2	forward GACTGCCGAGACTCCTACAA, reverse CTT-GTGGGTGAGGTAGATGG
Muc5ac	forward CTGGACCTGGAGGTTGTATG, reverse CAGTAGTGAGGGTT-GGATGG
Reg3b	forward ATACCCTCCGCACGCATTAGTTG, reverse CTCCATTCCCATCCAC-CTCCATTG
Reg3g	forward AAGGTGAAGTTGCCAAGAAAG, reverse CATTGCTCCAC-TCCCATCC
Tnfa	forward CGAGTGACAAGCCTGTAGCC, reverse AAGAGAACCTGGGAG-TAGACAAG
**Bacteria**	
Actinobacteria	forward TACGGCCGCAAGGCTA, reverse CGCGGCCTATCAGCTTGTTG
Bacteroidetes	forward GGARCATGTGGTTTAATTCGATGAT, reverse AGCTGACGACAAC-CATGCAG
Proteobacteria	forward CCGCAAGGTTAAAACTCAAAGGAA, reverse CAGA-CATGTCAAGGGTAGGTAAGG
BetaProteobacte-ria	forward GAGTGGCGAACGGGTGAGTAATA, reverse TCCGCGAG-CATGAGGTCTTG
Enterobacteri-aceae	forward CAGGTCGTCACGGTAACAAG, reverse GTGGTTCAGTTTCAG-CATGTAC
E. coli	forward CAAGTCATCATGGCCCTTAC, reverse CGGACTACGACGCAC-TTTAT
Firmicutes	forward GGAGYATGTGGTTTAATTCGAAGCA, reverse AGCTGACGACAAC-CATGCAC
Clostridiaceae	forward AGCGTTGTCCGGATTTACTG, reverse CGCTTAC-CTCTCCGACACTC
Lactobacillaceae	forward TGGATGCCTTGGCACTAGGA, reverse AAATCTCCGGATCAAA-GCTTACTTAT
L. reuteri	forward ACCGAGAACCACCGCGTTATTT, reverse CATAACTTAAC-CTAAACAATCAAAGATTGTCT

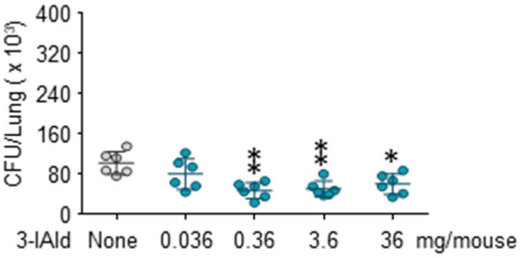

Figure S1. Dose-dependent activity of 3-IAld in vivo. C57BL/6 mice were infected with *Aspergillus* conidia and treated with 3-IAld at different doses intranasally. Fungal growth (Log_{10} CFU in the lung). Values represent the mean ± SD of 6 mice per group. None, infected mice. * $p < 0.05$; ** $p < 0.01$, One-way ANOVA - Bonferroni's, treated vs None mice.

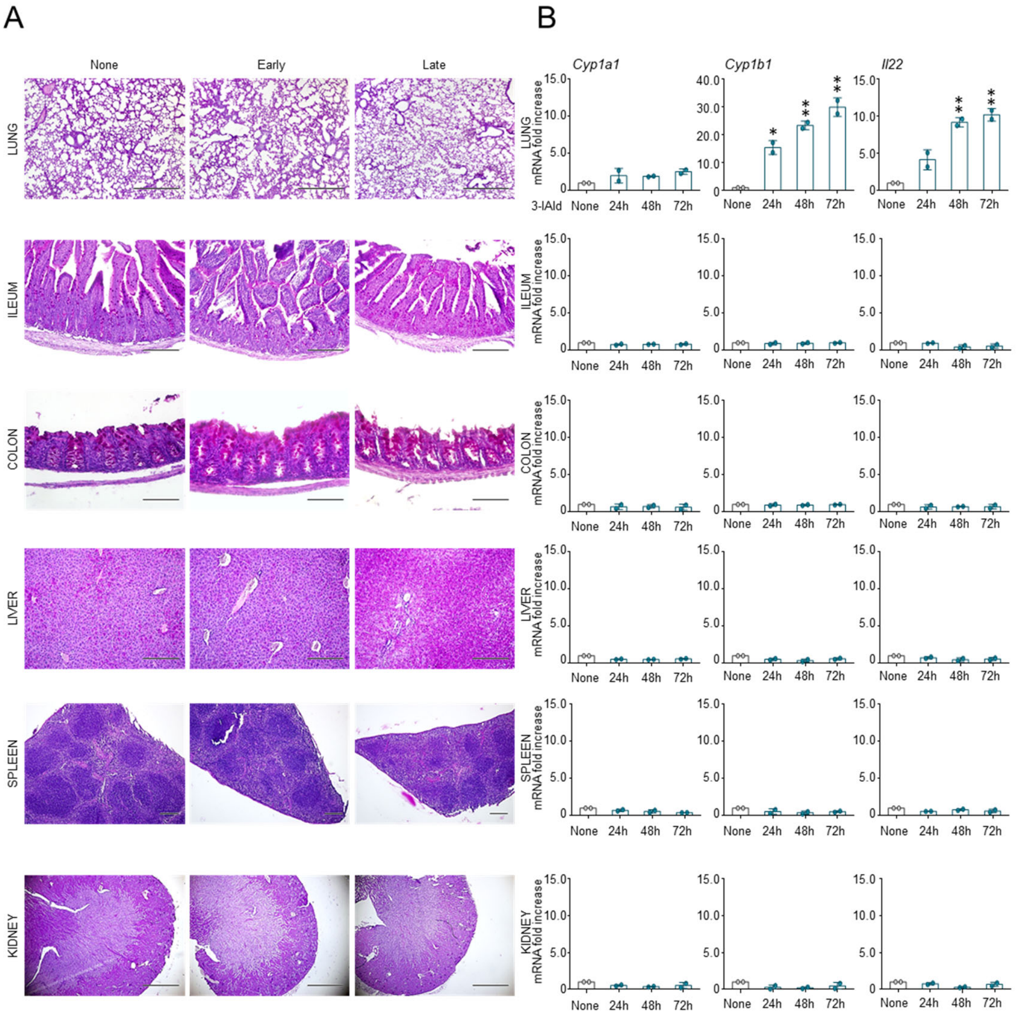

Figure S2. Lack of systemic toxicity and AhR activation in *Cftr* $^{F508del/F508del}$ mice administered with 3-IAld-PI. Mice received 4.5 mg/kg of 3-IAld directly delivered into the lung by pulmonary insufflation (PI) and evaluated for (A) signs of tissue pathology in the indicated organs 3 (early) and 7 (late) days later and (B) activation of AhR-target genes 24, 48 and 72 hours later. Photographs of PAS-stained sections were taken using a high-resolution Olympus DP71 microscope using an x10 objective. Scale bars, 100 (colon), 200 (ileum and liver), 400 (lung and spleen) µm and 1mm (kidney). (B) AhR-gene expression in total cells from the different organs by RT-PCR. Values represent the mean ± SD of 2 mice per group. * $p < 0.05$; **, $p < 0.01$, treated versus untreated mice (None); One-way ANOVA - Bonferroni's.
